# Dynamics Studies of Nitrogen Interstitial in GaN from Ab Initio Calculations

**DOI:** 10.3390/ma13163627

**Published:** 2020-08-17

**Authors:** Huan He, Wenbo Liu, Pengbo Zhang, Wenlong Liao, Dayin Tong, Lin Yang, Chaohui He, Hang Zang, Hongxiang Zong

**Affiliations:** 1 School of Nuclear Science and Technology, Xi’an Jiaotong University, Xi’an 710049, China; hehuan0422@stu.xjtu.edu.cn (H.H.); liuwenbo@xjtu.edu.cn (W.L.); lwl1551528661@stu.xjtu.edu.cn (W.L.); tdy8008208820@stu.xjtu.edu.cn (D.T.); zanghang@xjtu.edu.cn (H.Z.); 2 School of Physics, Dalian Maritime University, Dalian 116026, China; zhangpb@dlmu.edu.cn; 3 Department of Chemistry, University of Manchester, Manchester M13 9PR, UK; lin.yang-10@postgrad.manchester.ac.uk; 4 State Key Laboratory for Mechanical Behavior of Materials, Xi’an Jiaotong University, Xi’an 710049, China; zonghust@mail.xjtu.edu.cn

**Keywords:** ab initio calculation, nitrogen interstitial, migration, rotation, Frenkel pair

## Abstract

Understanding the properties of defects is crucial to design higher performance semiconductor materials because they influence the electronic and optical properties significantly. Using ab initio calculations, the dynamics properties of nitrogen interstitial in GaN material, including the configuration, migration, and interaction with vacancy were systematically investigated in the present work. By introducing different sites of foreign nitrogen atom, the most stable configuration of nitrogen interstitial was calculated to show a threefold symmetry in each layer and different charge states were characterized, respectively. In the researches of migration, two migration paths, in-plane and out-of-plane, were considered. With regards to the in-plane migration, an intermediated rotation process was observed first time. Due to this rotation behavior, two different barriers were demonstrated to reveal that the migration is an anisotropic behavior. Additionally, charged nitrogen Frenkel pair was found to be a relatively stable defect complex and its well separation distance was about 3.9 Å. Part of our results are in good agreement with the experimental results, and our work provides underlying insights of the identification and dynamics of nitrogen interstitial in GaN material. This study of defects in GaN material is useful to establish a more complete theory and improve the performance of GaN-based devices.

## 1. Introduction

Gallium nitride (GaN) with a wide-gap (~3.4 eV) is an excellent semiconductor material. Nowadays, it has been widely used in visible–UV light emitting devices (LEDs) [1], laser diodes (LDs), 5G communications, and high power/frequency transistors [2,3] because of its outstanding properties, such as high electron mobility and high breakdown voltage. Additionally, the high radiation tolerance [4,5] of GaN makes it a promising material for the application in satellites [6]. However, in the process of GaN growth, the large lattice mismatch of GaN with its substrate (silicon carbide (SiC) or sapphire) is inevitable. In addition, there are varieties of defects appearing in the whole fabrication process, including native point defects and impurities, such as C, O, H, as well as dislocations [7,8]. Therefore, it is still a big challenge to improve the performance of GaN-based devices.

The influence of defects in GaN material has been studied by experimental or theoretical work for years. With deep levels transient spectroscopy (DLTS), Kanegae et.al [9] observed that E_3_ (*E*_c_ −0.60 eV) and H_1_ (*E*_v_ +0.87 eV) are dominating traps in n-type GaN, and they attributed H_1_ trap to the gallium vacancy or C related defects. Sunay et.al [10] applied electron paramagnetic resonance (EPR) to find that the hole is around the Mg acceptor. In addition, by using positron annihilation spectroscopy (PAS), EPR, and density functional theory (DFT), Bardeleben et.al [11] found that the isolated N interstitial is unstable and prefers to form a split interstitial configuration, which is located at *E*_c_ −1.0 eV. However, experimental devices have limitations, i.e., DLTS can only obtain the energy level but not the specific type of defect.

Therefore, it leaves much room for theoretical simulation works, which can help us understand these defects from the atomic level. As an important complementarity, theoretical simulation is another efficient approach to understanding the property of defects. Among the theoretical work on GaN, varieties of studies [12,13,14,15,16,17] were put forward to obtain the transition levels related to the defects. They eventually concluded that N_i_ can occupy five charge states within the band gap. However, in some cases, such as under the doping process or radiation environment, the defects may move among the material and interact with other defects, instead of keeping as static ones. In contrast with abundant of calculations of static properties, less is known about the dynamics studies of defects [18,19], especially the interaction of defects.

In order to achieve a better understanding of defects in GaN material, in this paper, we performed ab initio calculations that focus on the nitrogen interstitial (N_i_). First, the most stable configurations of different charge states were calculated. Then, the migration of it was studied and a rotation process, which was not reported in previous researches, was included. Finally, its interaction with vacancy (V_N_) was systematically investigated.

## 2. Methods

Density functional theory calculations were performed with the Vienna Ab initio Simulation Package (VASP) 5.4.4 [20] code using pseudopotentials generated with the projector augmented wave (PAW) approach. The exchange-correlation function was treated with the generalized gradient approximation (GGA) using the parameterization by Perdew, Burke, and Ernzerhof (PBE) [21,22]. The valence electron configurations of 2s^2^2p^3^ for N and 3d^10^4s^2^4p^1^ for Ga were considered. The supercell method was applied with the periodic boundary conditions. Specifically, 4 × 4 × 2 supercell with 128 atoms was large enough to use in our simulations. The gamma k-point mesh was set to 2 × 2 × 2, and the cutoff energy was set as 500 eV. The convergence of force was less than 0.01 eV/Å for all the computations, while the criterion of energy was minimized to a value of 10^−5^ eV. Climbing image nudged elastic band (CI-NEB) method [23] was used to investigate the migration processes and their corresponding possible minimum energy path, as well as the recombination process. Furthermore, in order to obtain an accurate transition state faster, DIMER method [24] was applied sometimes within CI-NEB method. The criterions of energy and force applied in these two methods were the same as the values in the optimization of structures.

**Figure 1:**
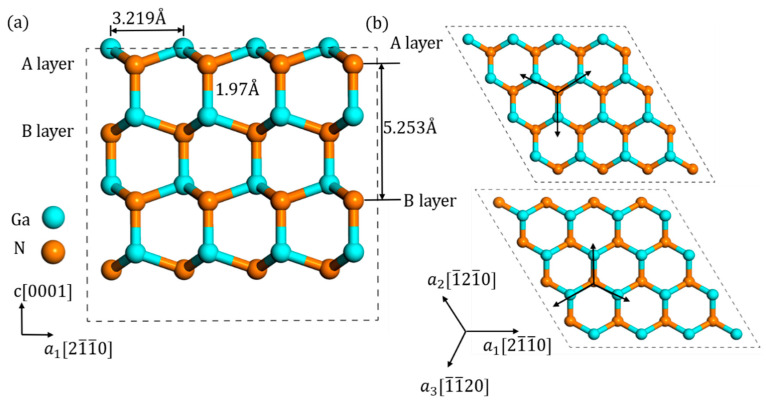
Different view of the structure of wurtzite GaN material: (**a**) side view and (**b**) top view of A layer and B layer.

The model of GaN crystal was based on the Material Project database [25], and after structure optimization, lattice parameter was eventually optimized to a = 3.219 Å and c/a = 1.632, which matched well with the previous experiments [26]. As shown in Figure 1a, every N atom was bonded with four Ga atoms and the bond length is 1.97 Å. Additionally, there are two distinct N layers in a prefect GaN crystal, named “A” and “B” layer, characterized by the orientation of N atom with the nearest Ga atoms. As shown in Figure 1b, the orientations of A layer were opposite to those of B layer.

The formation energy of a charged N_i_ is described as follows Equation (1) [27]:

(1) $$E_{f}\left[N_{i}^{q}\right]=E_{\mathrm{tot}}\left[N_{i}^{q}\right]-E_{\mathrm{tot}}\left[\mathrm{bulk}\right]-\mu_{N}+q\left(E_{F}+E_{g}\right)+E_{\mathrm{corr}}^{q}$$

where $E_{\mathrm{tot}}\left[N_{i}^{q}\right]$ is the total energy of supercell with charged defects, while $E_{\mathrm{tot}}\left[\mathrm{bulk}\right]$ is the total energy of system without defects. $\mu_{N}$ is the chemical potential of the N element. Additionally, $E_{F}$ is the Fermi level, which is regarded as a variable, ranging from 0 to the band gap value $E_{g}$. Additionally, the last term is a correction term of the supercell system.

## 3. Results

### 3.1. Configurations of N_i_

**Figure 2:**
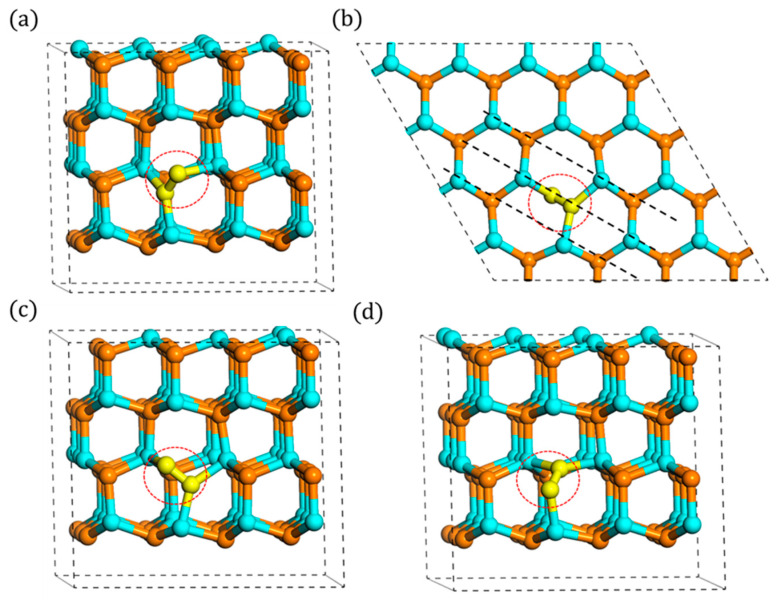
Configurations of split interstitial: (**a**) side view of A_1_, (**b**) top view of A_1_, (**c**) side view of A_2_, and (**d**) side view of A_3_ (N_i_ atoms are denoted by yellow spheres, the same below).

With regards to typical interstitials in hexagonal close packing (hcp) type materials, two types of high symmetry sites are the most common ones: the tetrahedral (T-site) and octahedral (O-site) sites. The T-site has four nearest neighbors and the O-site has six nearest neighbors. Therefore, it was first required to determine the most stable configuration of N_i_. Tens of (more than 50) possible sites were carried out assuming that N_i_ could locate in any possible space in the crystal. The results revealed that wherever the initial N_i_ is, even the O or T sites, it always prefers a split interstitial configuration. This configuration is a N-N dimer tilted bond as presented in Figure 2, and the calculated formation energy of the neutral state is 4.67 eV. These findings correspond to the previous researches [12,14,17] well. Furthermore, some new specific characteristics of these N_i_ were concluded, and they could be directly applied in later simulations or experiments.

**Figure A1:**
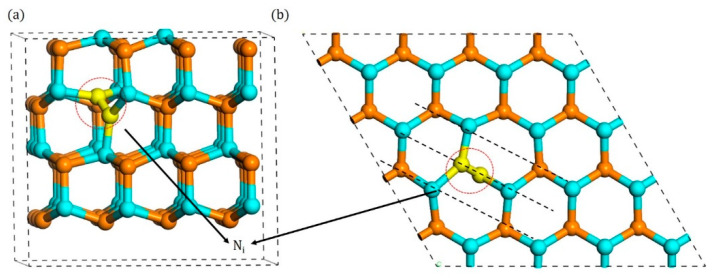
Configurations of B_1_ type split interstitial: (**a**) side view and (**b**) top view.

**Figure A2:**
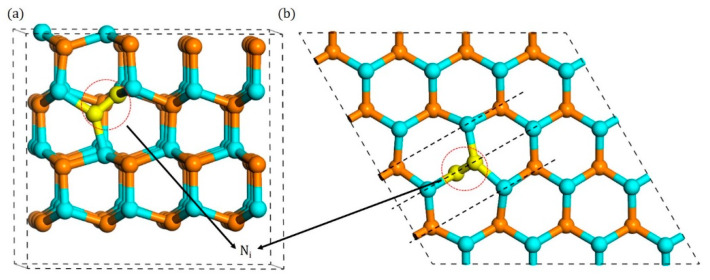
Configurations of B_2_ type split interstitial: (**a**) side view and (**b**) top view.

**Figure A3:**
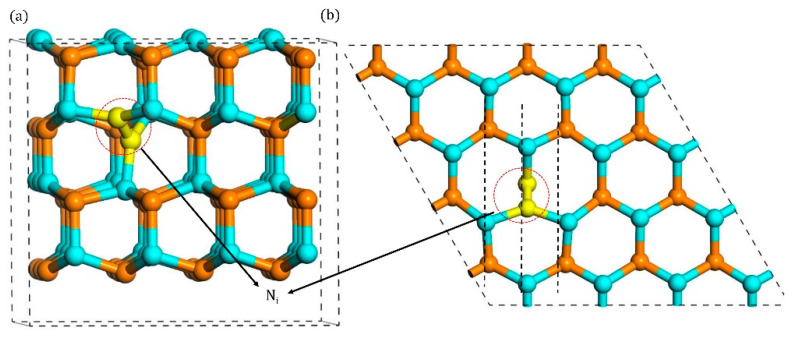
Configurations of B_3_ type split interstitial: (**a**) side view and (**b**) top view.

Because of the high symmetry of wurtzite structure, as shown in Figure 1, the tilted N_i_ should be sixfold symmetry. However, due to the different orientations of N atom with its nearest Ga atoms, the tilted N_i_ presents a threefold symmetry in each layer, respectively, not a true sixfold symmetry. These threefold symmetry configurations can transform to each other by rotating [0001] axis by multiples of 120°. The example of three configurations on A layer, named “A_1_,” “A_2_,” and “A_3_” is shown in Figure 2, and the following discussions are mainly based on this A_1_ configuration. Note that the foreign atom can become either atom in this dimer bond. Due to the tilting characteristic, its direction’s projection on the c-plane is the same as that between the origin N atom with the nearest Ga atom, which is [1−10c] (parameter c is about 1.75). Additionally, its bond length is measured about 1.35 Å. This N-N bond length is a little larger than N_2_ gas molecule (1.11 Å) and much less than normal Ga-N bond length (1.97 Å), which indicates that it may be a relatively stable bond. The schematics of configurations on B layer are included in Figure A1, Figure A2 and Figure A3 in Appendix A.

As discussed in other studies [12,13,18,27], N_i_ always occupies five charge states (−1 to 3) within the band gap. Therefore, the charge state of −1 to 3 was considered in our research. With regards to different charge states, the bond length of N_i_ changes significantly, from 1.44 to 1.15 Å. However, the directions are like the neutral ones. The differences between them mainly reflect on the parameter c, and the corresponding results are listed in Table 1. Comparing the values in Table 1, the bond length of N_i_ was found to increase as the charge state decreases, indicating that positive charge states may provide a more attractive force than negative charge states. On the other hand, our calculated bond length is consistent well with previous work [18,27].

### 3.2. Migration of N_i_

**Figure 3:**
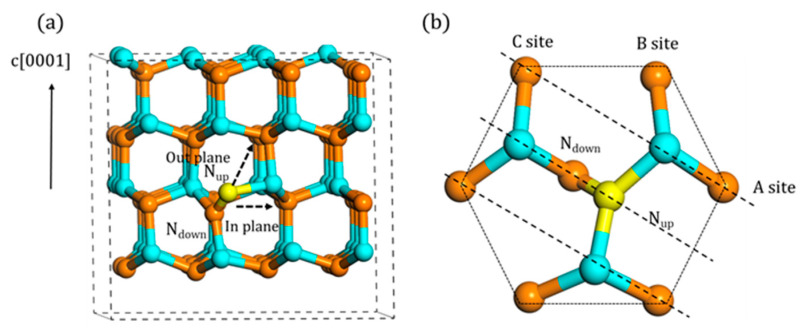
The migration mechanism of A_1_ type N_i_ atom (the yellow atom is migrated N atom, the same as below): (**a**) two possible paths and (**b**) the top view of the in-plane migration.

Because of the lower formation energy among the simple point defects, N_i_ is one of the most common native defects in the GaN material. Therefore, its migration behavior influences the properties of material significantly. As shown in Figure 3a, there are two possible migration paths, in-plane and out-of-plane. Only the first nearest neighbor (1NN) atoms were considered here. With regards to six nearest atoms in-plane, determined by the orientation of N_i_, the migration could be divided into three categories, A, B, and C site, as shown in Figure 3b. Since C site is symmetrical with A site, only A and B site was taken into consideration. Furthermore, the upward one (N_up_) in N_i_ was regarded as the atom to migrate.

#### 3.2.1. In-Plane Migration

**Figure 4:**
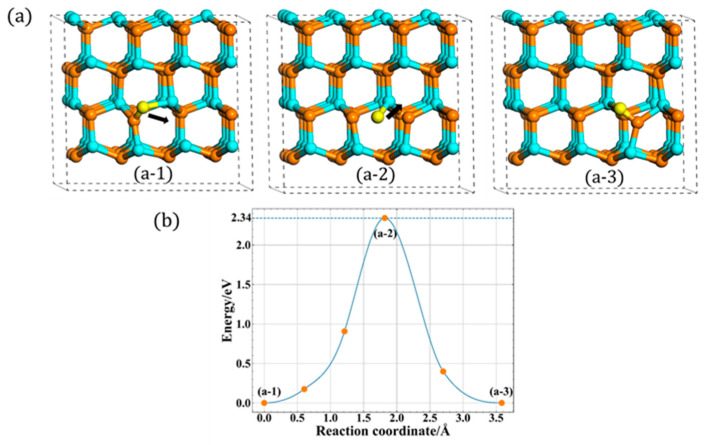
The direct in-plane migration for A site: (**a**) the process of direct migration. (**b**) The corresponding energy barrier (the solid dots are interpolated points and the solid line show the energy path obtained from CI-NEB methods).

With regards to the A site, two different migration mechanisms of N_i_ are found. The first one is a direct migration, the N_up_ atom first breaks the bond of interstitial and then directly migrates to the A site, forming a new type N_i_. The corresponding migration process is shown in Figure 4a. Note that the initial state (a-1) is different from the final state (a-3), which is another type of orientation in this layer. Furthermore, its related energy barrier is shown in Figure 4b, and the migration barrier is 2.34 eV, which is consistent well with previous studies, i.e., 2.33 eV [14] and 2.4 eV [17,18].

**Figure 5:**
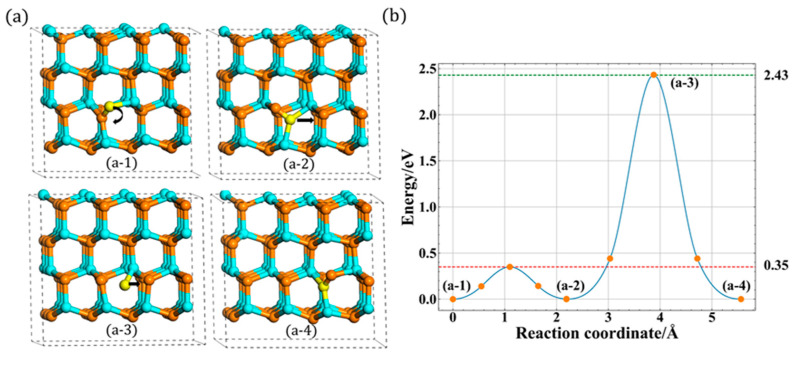
The indirect in-plane migration for A site: (**a**) the process of indirect migration and (**b**) the corresponding energy barrier.

Compared with the previous studies [16,17] of migration, a new indirect migration was observed in our study, as shown in Figure 5a. In this indirect process, the N atom’s migration behavior is a little different while a rotation behavior occurs first during the whole migration process. First, it rotates around the c-plane with relatively low energy barrier, about 0.35 eV. During the rotation process, the bond length increases first and then decreases to the stable configuration, which is another type of configuration (A_2_) in this layer. After the rotation process, the N atom starts to move and then migrates to A site. Compared with the direct migration, there are two different points. First, the final state (a-4) is the same as the initial state (a-1) before rotation. Second, the migrated atom transfers finally to the lower site after the whole migration. The whole process is presented in Figure 5b, and the migration barrier is 2.43 eV, slightly larger than the first type of migration path. And the animations of these two migration are included in Figures S1 and S2 in Supplementary Materials.

**Figure 6:**
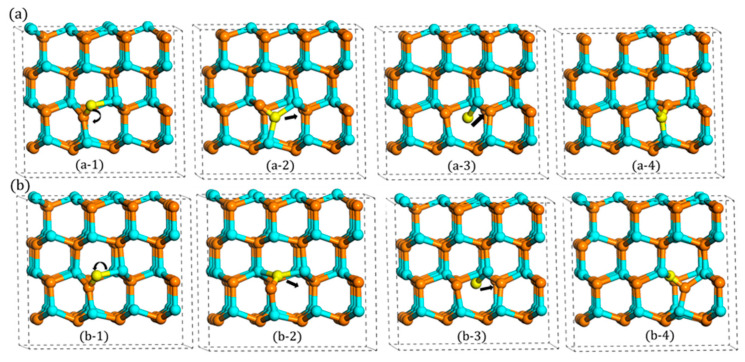
Two rotation-migration mechanisms to B site: (**a**) A_1_ rotates to A_2_ and (**b**) A_1_ rotates to A_3_.

However, this little difference (2.34 vs. 2.43 eV) is not actually influenced by the rotation behavior, because these different migration barriers were also observed when the N atom migrates to B site with two different indirect migrations. With regards to B site, the N atom cannot migrate directly like the case of the A site, since B site is far away from the A_1_ type’s orientation. Therefore, it must rotate first to A_2_ or A_3_ type, and then, it migrates directly to B site. When it starts to migrate from A_2_ type, the migration barrier is 2.43 eV, the same as the indirect migration to A site. However, when it rotates first to A_3_ type, this process only needs 2.34 eV, which is equivalent to the direct migration. This phenomenon, as shown in Figure 6, illustrates that the different migration barriers (2.43 vs. 2.34 eV) are not influenced by the rotation process. In addition to this, it also can be seen that when the N atom presents downward migration, the migration barrier is 2.43 eV, whereas when the N atom migrates upward, the value changes to 2.34 eV.

**Figure 7:**
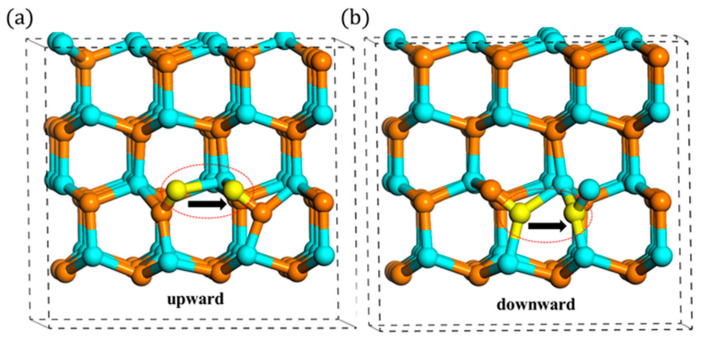
(**a**) Upward and (**b**) downward migration (the left yellow atom migrates to the right yellow atom).

In summary, the in-plane migrations actually have two paths, upward and downward ones. Additionally, they can transform to each other by the rotation behavior. The schematic is shown in Figure 7. Additionally, as shown in Table 2, under high charge states, the difference of migration barriers is a little large. Therefore, by introducing the consideration of rotation behavior of N interstitial, it is found that the in-plane migration of N atoms is an anisotropic behavior, which is different from the conclusions of previous studies [16,17].

#### 3.2.2. Out-of-Plane Migration

With regards to out-of-plane migration of N_i_, the results are like previous work [16,17] and show that it could be a direct migration as discussed above. All of the detailed values are also listed in Table 2.

The data in Table 2 indicates that the rotation and migration barriers vary in the different charge states and part of our results are consistent well with previous work [14,18,19]. The results show that the differences between the upward and downward paths in plane are due to the rotation barrier. Additionally, the migration barriers of in-plane and out-of-plane are also different. However, previous studies about migration of N_i_ did not observe the rotation behavior and concluded that it is an isotropic behavior with the same energy barriers from the in-plane to out-of-plane [18]. In our detailed researches, although the rotation barrier of 0.04–0.31 eV is very small compared with migration barriers of about 2 eV, its existence shows that the migration of N_i_ is not the same, especially in the migration mechanisms. Additionally, under room temperature, according to the Arrhenius equation, the difference of 0.1 eV may cause several orders of magnitude difference of gigahertz, which cannot be neglected in the application of high frequency devices [28,29]. Due to this little barrier, the migration process of N_i_ can present different possibilities, not merely a direct migration, and the rotation behavior provides a new perspective of the migration of N_i_ by the theoretical work. Therefore, the migration barrier is concluded to be an anisotropic behavior by the calculations about different migration barriers.

### 3.3. Stability of N_i_-V_N_ Complex

Frenkel pair, as a common type of defect pair in material, plays an important role in the material property, especially under irradiation or ion modification. However, there are a few studies about this type of defect pair in GaN material, especially the theoretical work. Furthermore, the formation energy of neutral one is 6.82 eV, a little larger than the value of the isolated one, meaning that N Frenkel pair is easier to appear. Therefore, the stability of N Frenkel pair (N_i_-V_N_) was investigated here and the charge states of 0 to +3 were considered due to the possible charge states of isolated defects. Since N_i_ is a tilted configuration, a range of initial distances (*d*_FP-id_) of N_i_-V_N_ were considered for different charge states. Additionally, the separation distance (*d*_FP-sd_) is defined as the nearest distance of the final N_i_-V_N_.

As expected, due to the large bond length of N_i_, the recombination (annihilation of interstitial and vacancy, N_i_ + V_N_→N_N_) is not observed for variety of N_i_-V_N_, except the neutral one with the nearest *d*_FP-id_ (2.75 Å) (in the following discussions, this specific condition is not considered). This phenomenon indicates that N_i_ is a type of relatively stable defect since it cannot nearly recombine spontaneously. In addition, consequently, N_i_ and V_N_ remain to locate their site and the N_i_ bond does not break itself. The bond lengths of N_i_ in N_i_-V_N_ are illustrated in Table 3. Compared with the values in Table 1, the bond length of N_i_ in (N_i_-V_N_)*^q^* (0 ≤ *q* ≤ 3) is found to be almost equal to it of (N_i_)*^q^*^−1^. Accordingly, (N_i_-V_N_)*^q^* is assumed to consist of (N_i_)*^q^*^−1^ and (V_N_)^+1^. In order to verify this point more accurately, a more detailed charge analysis of these defects, containing N_i_-V_N_, isolated N_i_ and V_N_, is required to be carried out.

Bader charge analysis [30,31] is used to investigate the charge transfer between atoms quantitatively. With regards to the perfect material, each N atom gains 1.54|e| from its four nearest Ga atoms, vice versa. However, when the system contains N_i_ defects, this value changes a lot. For the neutral one, the charges of two N_i_ atoms are identical to become 0.87|e|, and other Ga and N atoms in this system almost do not change. This phenomenon shows that due to the introduction of N_i_, the charge of one original N atom decreases and this decrement mainly transfers to the foreign N atom. Since the charges of two N_i_ atoms are identical, their average values are adopted in the comparison. As for V_N_, due to its special property without atoms, the average charges of the four nearest Ga atoms are considered instead. The comparisons between them are shown in Table 4.

Compared with the Bader charge of N_i_-V_N_ and isolated defects, the Bader charges of N_i_ and V_N_ in (N_i_-V_N_)*^q^* are consistent well with the values of isolated ones, e.g., (N_i_-V_N_)^+2^ (0.67/1.21 |e|), (N_i_)^+1^ (0.66 |e|) and (V_N_)^+1^ (1.21 |e|). This accurate comparison suggests that for the N_i_-V_N_ in the q charge state, this configuration could be associated with the reaction of (N_i_)*^q^*^−1^ and (V_N_)^+1^, namely, (N_i_-V_N_)*^q^* →(N_i_)*^q^*^−1^ + (V_N_)^+1^. The charge state of V_N_ always remains the constant value of +1 in N_i_-V_N_ pair. This similar relationship is also found in Si material [32], however, in their studies, they observed that Si interstitial is the defect that remains as constant charge. Additionally, this phenomenon may explain that why only the neutral nearest N_i_-V_N_ recombine spontaneously, since the neutral N_i_-V_N_ is consist of (N_i_)^−1^ and (V_N_)^+1^ and they are exactly opposite charge.

Therefore, as mentioned above, the binding energy, which is often used to evaluated the stability of defect complex, is defined as Equation (2):

(2) $$E_{b}{=(E}_{{\left(N_{i}{\text{-}V}_{N}\right)}^{q}}+E_{\mathrm{perfect}}{)-(E}_{{\left(N_{i}\right)}^{q-1}}+E_{{\left(V_{N}\right)}^{+1}})$$

**Figure 8:**
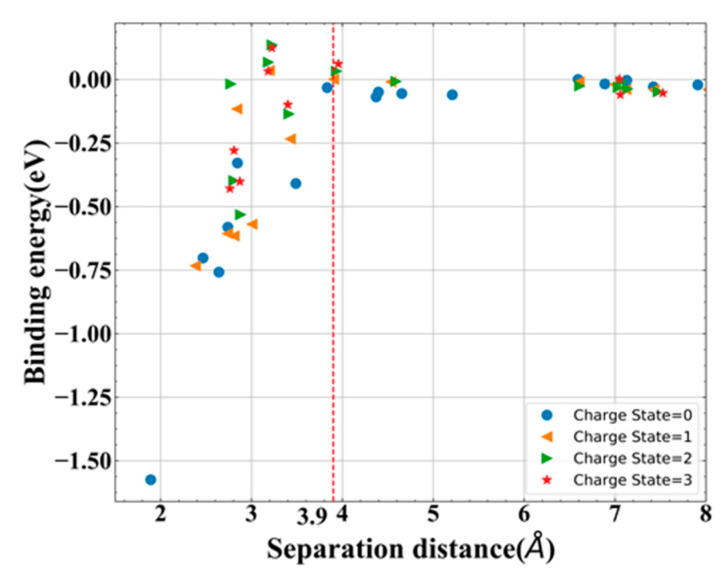
Binding energy of different charge states of N_i_–V_N_ as a function of *d*_FP-sd_.

Note that *E*_b_ < 0 means attraction and *E*_b_ > 0 means repulsion for this definition. By introducing different *d*_FP-id_ of N_i_-V_N_, the binding energies with different *d*_FP-sd_ were obtained in Figure 8. From this figure, the binding energy of N_i_-V_N_ was observed to be influenced by the *d*_FP-sd_. As the *d*_FP-sd_ increases, the binding energy increases first and then stays at about 0 eV from 3.9 Å, which indicates that the well-separated distance of N_i_-V_N_ is 3.9 Å.

Besides that, with the help of NEB method, the recombination barrier of the well-separated neutral N_i_-V_N_ (*d*_FP-sd_ = 3.9 Å) is found to be 2.1 eV, which also verifies that this distance is a truly well separation distance from another perspective. It is worth noting that this recombination barrier is a little less than the migration barrier. Furthermore, the nearest neutral (*d*_FP-sd_ = 1.89 Å) one is also calculated to be 0.15 eV. Such small barrier value indicates that this distance of N_i_-V_N_ is a metastable defect complex with strong binding, and it is easier to annihilate under ambient temperature.

### 3.4. Comparisons with the Experiments

A previous EPR experimental study [11] reported that N_i_ starts to anneal at 673 K during the first annealing stage, and this process of annealing may relate to the migration process. Additionally, the experiments also showed that this N_i_ locates at the Fermi level 1 eV below the conduction band minimum, indicating that it most likely corresponds to −1 charged state. Therefore, in order to verify our computation results, the reported experimental results were implemented to compare. According to the previous studies [33,34] of the correlation between activation energy and temperature, Arrhenius law was applied to the activation energy in most of cases. Thus, based on the Equation (3) of Arrhenius law, the activation energy, namely, the migration energy, is given by:

(3) $${k=Ae}^{-\frac{E_{a}}{k_{B}T}}$$

where *k* is the rate constant, which is always regarded as 1 Hz [34]. Additionally, the pre-exponential factor *A* is always represented by the material’s Debye frequency of 13 THz [35]. Therefore, according to the above parameters, the migration energy in the experiment is approximately calculated as 1.75 eV. In addition, with regards to our calculations, as can be seen from Table 2, the migration energy of N_i_ with −1 charge state is 1.80 eV, which is in good agreement with the experimental results and closer than other works [18,19]. In other words, this comparable result provides another perspective to prove our results.

## 4. Conclusions

In the present work, ab initio calculations have been performed to investigate the defect of nitrogen interstitial (N_i_) in GaN material. With regards to different N_i_ configurations, it is found that the most stable configuration of N_i_ presents a threefold symmetry in each layer and different charge states of N_i_ show a similar orientation but different bond lengths.

For the nearest in-plane migration of N_i_, a rotation process which has not been reported is observed. Due to this process, the in-plane migration is found to be divided into two paths, upward and downward paths and the migration barriers of them differ at some charge states. Different from previous studies, our specific work shows that the migration of N atom is an anisotropic process, especially in the migration mechanisms. Furthermore, part of our results of migration of N_i_ are consistent well with the existing experiments and the corresponding theories.

As for charged N Frenkel pair, it is found to be a relatively stable defect complex as expected. According to the results of bond length and charge analysis, the charged complex (N_i_-V_N_)*^q^* may consist of (N_i_)*^q^*^−1^ and (V_N_)^+1^. Additionally, the calculations of binding energy and recombination barrier also reveal that the well separation distance of N_i_-V_N_ is about 3.9 Å.

The calculated results are useful to reveal some mechanisms of point defect in GaN, i.e., rotation behavior and charged Frenkel pair. This work also provides helpful perspectives in the identification and dynamics studies of GaN material by a theoretical sight.

## Figures and Tables

**Table 1:** Bond length and parameter c of different charge states of N_i_.

Charge State	*d*_N-N_ (Å)			Parameter c
	This Work	Other Work	Other Work (%)	
3	1.15	1.12 ^a^ 1.11 ^b^	2.61 ^a^ 3.48 ^b^	1.50
2	1.20	1.18 ^a^	1.67 ^a^	1.51
1	1.27	1.25 ^a^	1.57 ^a^	1.55
0	1.35	1.34 ^a^	0.74 ^a^	1.75
−1	1.44	1.45 ^a^ 1.41 ^b^	0.69 ^a^ 2.08 ^b^	2.27

^a^ Reference [19]; ^b^ Reference [17].

**Table 2:** Rotation and migration barriers for different charge states of N_i_.

Charge State	Rotation Barrier/eV	In/eV(upward/downward)	Out/eV	In/eV	Out/eV
−1	0.33	1.80/1.80	1.87	1.9 ^a^, 1.6 ^b^	1.9 ^a^, 1.6 ^b^
0	0.35	2.34/2.43	2.40	2.4 ^a^, 2.4 ^b^, 2.33 ^c^	2.4 ^a^, 2.4 ^b^
1	0.27	2.48/2.52	2.18	2.2 ^a^, 2.1 ^b^	2.1 ^a^, 2.1 ^b^
2	0.24	1.98/2.08	2.13	2.1 ^a^, 2.5 ^b^	2.2 ^a^, 2.5 ^b^
3	0.23	1.57/1.26	2.13	2.1 ^a^, 1.4 ^b^	1.7 ^a^, 1.4 ^b^

^a^ Reference [19]; ^b^ Reference [18]; ^c^ Reference [14].

**Table 3:** Bond lengths of N_i_ in the nearest N_i_-V_N_ for different charge states.

Defect Type	Charge State	Bond Length/Å
N_i_-V_N_	+3	1.21
	+2	1.26
	+1	1.34
	0	1.44

**Table 4:** Bader charges of N_i_, V_N_ in the nearest N_i_-V_N_ and isolated N_i_, V_N_.

Defect Type	Charge State	N_i_-V_N_ (N_i_/V_N_)	Isolated One (N_i_/V_N_)
Bader Charge(|e|)	3	0.51/1.23	0.21/1.43
	2	0.67/1.21	0.44/1.28
	1	0.84/1.19	0.66/1.21
	0	1.04/1.22	0.87/1.15
	−1	–	1.05/1.07

## Supplementary Materials

The following are available online at https://www.mdpi.com/1996-1944/13/16/3627/s1. Figure S1: Direct migration; Figure S2: Indirect migration.
