# Supplementary figures and images for: Dynamics Studies of Nitrogen Interstitial in GaN from Ab Initio Calculations
Source: Materials (Basel). 2020 Aug 17;13(16):3627. doi: 10.3390/ma13163627 (PMC7475889; doi:10.3390/ma13163627)

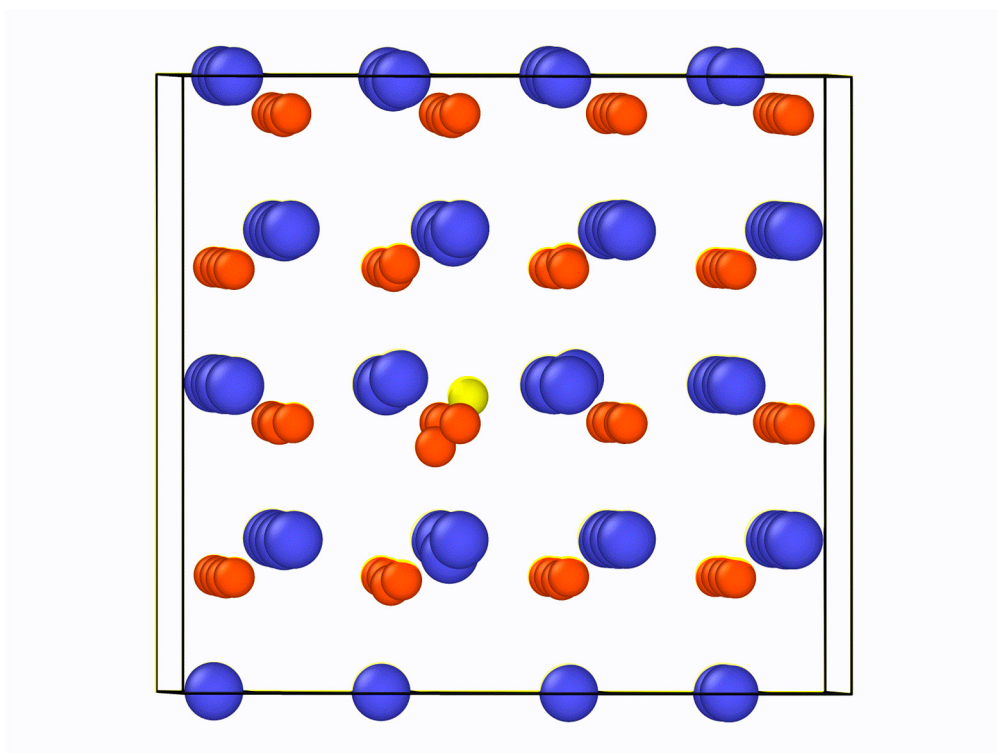

**Figure S1.** Direct migration.

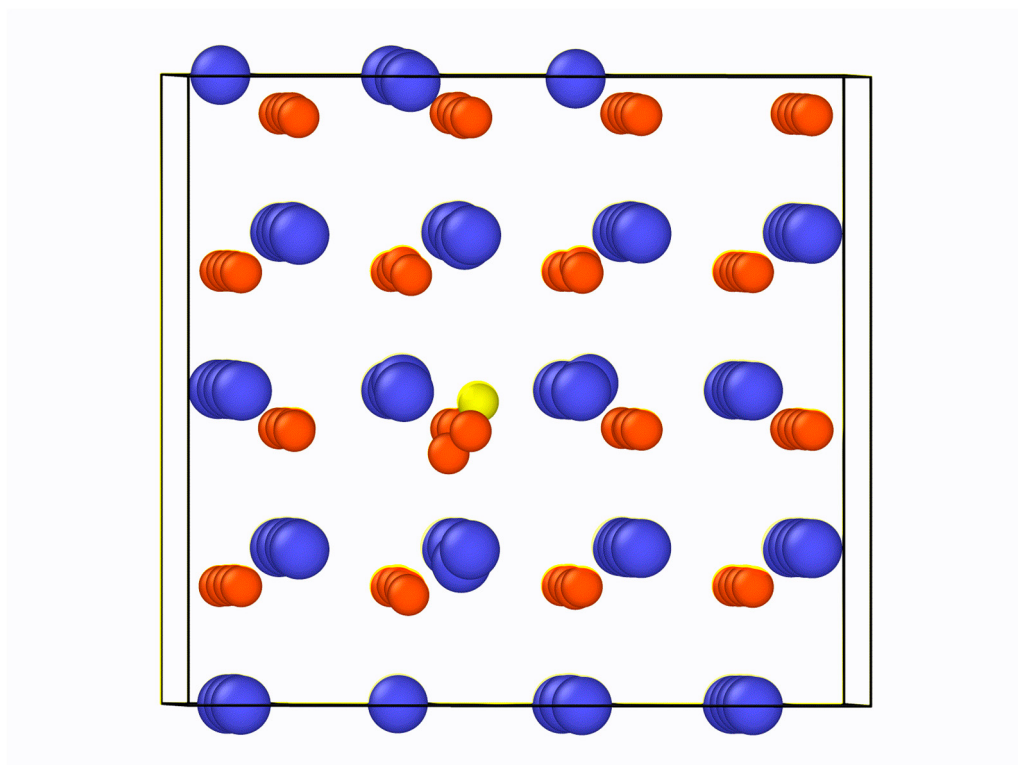

**Figure S2.** Indirect migration.

Supplement: Supplementary file 1 [file materials-13-03627-s001.pdf]
